# Multifaceted Empathy Test (MET): Validity evidence for the Brazilian population concerning the computer-based (face-to-face) and online versions

**DOI:** 10.1371/journal.pone.0284524

**Published:** 2023-07-13

**Authors:** Felipe Fernandes Vieira de Lima, Giordano Rossi, Rafael Guimarães dos Santos, Flávia de Lima Osório

**Affiliations:** 1 Medical School of Ribeirão Preto, São Paulo University, Ribeirão Preto, SP, Brazil; 2 National Institute of Science and Technology (INCT-TM, CNPq), Brasília, Brazil; University of Sao Paulo, School of Medicine, BRAZIL

## Abstract

**Background:**

The lack of empathy is associated with several psychological and behavioral disorders, and it is important to assess this construct broadly, through multi-methods.

**Objective:**

To conduct a psychometric analysis of the Brazilian version of the Multifaceted Empathy Test (MET), a computerized task that assesses emotional and cognitive empathy.

**Methods:**

The samples were recruited from the community using the snowball method (phase 1: face-to-face; N = 142) and through social media (phase 2: online; N = 519). The participants completed the MET and the Interpersonal Reactivity Index (IRI) to assess the convergent validity between the instruments. To assess validity with correlated constructs (resilient coping and stress), the Brief Resilient Coping Scale and Perceived Stress Scale were used. A task was also implemented in the face-to-face application to assess facial emotions. The retest was applied 25 days later to a portion of the sample (face-to-face: N = 31; online: N = 102).

**Results:**

It was observed adequate test-retest reliability for most items (ICC = 0.49–0.98), satisfactory infit and outfit indexes, discriminatory ability between sexes, weak convergent validity with empathy measures (r = 0.17–0.36), and correlate constructs (r = 0.12–0.46). MET presented good psychometric indicators, confirming its use in face-to-face/computer-based and online formats in clinical and research contexts. However, weaknesses were found regarding the cognitive subscale, demanding future studies to address larger samples to enable more robust conclusions concerning its adequacy. Further research on the instrument’s internal structure can also contribute to its improvement.

## Introduction

Empathy is a fundamental element of human experience [1], considered a domain of social cognition. It concerns the ability to understand and feel what someone else thinks and feels in affectively demanding situations and properly communicate such an understanding and feeling [2]. According to Baron-Cohen et al. [3], it is a multidimensional construct comprising a cognitive and an emotional component.

Lack of empathy is associated with various psychological and behavioral disorders, such as autism [4], personality disorders [5–7], social anxiety [8], and emotion regulation [9], among others. Additionally, Bordin et al. [10] point out relationships between anxiety and depression symptoms and declined empathy among health professionals. On the other hand, Spilg et al. [11] note that empathy is associated with favorable outcomes such as resilience among medical students.

Thus, systematically assessing empathy in clinical and research contexts is increasingly relevant. According to Lima & Osório [12], many different methods are used for this purpose, such as physiological measurements (e.g., skin conductivity and temperature, heart and respiratory rate), facial expressions and gestures analysis, standardized instruments based on interviews and self-assessment scales. The authors also note that in recent years, self-report measures have been the most frequently studied and used; though, some limitations associated with these measures are currently highlighted, such as social desirability bias. Thus, different resources based on multi-methods are increasingly needed to encompass the entire construct [13]. Emphasis has also been given to instruments with high ecological validity, such as computer-based instruments, which assess psychological phenomena more realistically because they provide multimedia resources such as images and videos that portray everyday situations [14].

From this perspective, Dziobek et al. [15] developed the Multifaceted Empathy Test (MET), an instrument using photorealistic stimuli to measure cognitive and emotional empathy simultaneously, which is one of its advantages, in addition to favoring greater ecological validity, less bias in the application and emission of responses. Furthermore, as it is a computerized instrument, it favors faster and more standardized application and correction The MET was originally published in German, but has also been translated and cross-culturally adapted into Chinese [16] and English [17], demonstrating adequate psychometric properties (e.g., reliability, internal consistency). Recently, it was culturally adapted to Brazilian Portuguese, and its content validity was assessed [18]. Despite being widely adopted by scientific studies [19–21], other types of validity and reliability remain to be assessed, in order to ensure the adequacy of the measure.

It is also noteworthy that in the current scenario, the use of advanced technologies that allow remote/online assessment of skills and other psychological and behavioral characteristics is increasingly necessary/desired. This fact encourages the development and/or adaptation of instruments to meet this demand, as well as the development of specific psychometric studies. For use in the Brazilian population, as pointed out by a review conducted by Lima and Osório [12], few instruments for assessing empathy are available (Empathy Inventory, Interpersonal Reactivity Index and Online Empathy Questionnaire), all of which are self-reported, based on items scored on Likert-type scales.

Therefore, this study’s objective is to present a psychometric assessment of the MET Brazilian version in the computer-based (face-to-face) and online versions, verify reliability indicators (i.e., internal consistency and test-retest), validity (based on external measures: known-group and convergent validity), and item response (difficulty and performance deviations based on the Rasch model). With this, we intend to add new psychometric evidence of the MET, assess its suitability for use in an online format and expand the resources available for the assessment of empathy in the Brazilian context.

## Materials and methods

This observational study has a psychometric approach. The psychometric properties of MET were assessed at two points in time. First, the psychometric indicators of the computer-based version applied computer-based (face-to-face) were tested and then, the instrument was adapted to be applied online using the REDCap software. Data collection took place between July 2020 and October 2021 (for more information on data collection see S1 Table).

The study was approved by the Research Ethics Committee of the *Hospital das Clínicas da Faculdade de Medicina de Ribeirão Preto da Universidade de São Paulo* (CAAE n° 05370818.9.0000.5440) and the subjects gave their consent in writing or by providing their digital signature on the Redcap platform.

### Participants and procedures

The sample size was based on the recommendations proposed by MacCallum et al [22]. For these authors, when the commonalities are high on average and the factors well determined, sample sizes in the range of 100 to 200 should be adequate to adequately estimate the population parameters. Inclusion and exclusion criteria were adopted to recruit two community samples to participate in each phase.

Inclusion criteria were: adult individuals (≥18 years old), both genders, literate, capable of reading and understanding texts, and voluntarily agreeing to participate in the study. Not completing any instrument or not finishing data collection were the criteria for excluding participants.

S1 Fig includes the flowchart presenting each sample’s composition. The final convenience samples comprised 142 participants (computer-based/face-to-face) and 519 participants (online).

The snowball sampling technique [23] was used to recruit the participants for computer-based (face-to-face) collection phase. It is a technique that uses chains of reference and indications. Briefly, key informants help the researcher to initiate recruitment. Then, the people indicated by these informants are asked to indicate new potential participants, with the desired characteristics, from their own personal network, and so on, until the desired sample is reached. First, data were collected using the following instruments (paper-and-pencil versions):

*Sociodemographic and clinical questionnaire*, composed of 11 items and developed for this study to collect complementary data concerning the participants’ sociodemographic and clinical information (gender, age, education, number of children, marital status, live together, religion, job, health problem, medication use and psychiatric diagnosis).

*Interpersonal Reactivity Index (IRI) is an* instrument that assesses emotional and cognitive empathy. It was proposed by Davis [24] and translated, adapted and psychometrically evaluate to Brazilian Portuguese by Koller et al [25] (α = 0.75; convergent validity = 0.54). Only three of the original scales compose the Brazilian version (Empathic Concern, Personal Distress, and Perspective Taking);

*Brief Resilient Coping Scale (BRCS)* is an instrument proposed by Sinclair and Wallston [26] to assess the use of coping strategies to solve problems in stressful situations. The version translated and adapted by Ribeiro and Morais [27] was used here, and demonstrated internal consistency (α = 0.53) and unifactorial structure.

*Perceived Stress Scale (PSS)* was proposed by Cohen et al. [28] and translated and adapted by Luft et al. [29]. It was designed to assess the individuals’ perception of how unpredictable and uncontrollable were the life events they experienced in the last month, providing a measure of stress perception.

In sequence, the computerized tasks were administered:

*a) Multifaceted Empathy Test*, developed by Dziobek et al. [15] and adapted by Foell et al. [17] to assess empathy’s cognitive and emotional aspects. It is composed of 40 photographs of people experiencing positive or negative situations in which the participants are asked to assess the photographs and answer two specific questions. In the first question, *“What emotion is this person feeling*?” the respondent may choose the alternative (out of four) that best represents the feeling elicited. The second question, *“How much do you empathize with this person*?*”* requires the participant to assess how much s/he feels empathically mobilized by the feeling the photograph expresses, using a Likert scale ranging from 1(not at all) to 9 (extremely). The stimuli are presented in eight blocks with ten photographs each.

*b) Facial Emotion Recognition Task* (FERT) [30]: to assess the recognition of dynamic facial emotions. A series of 24 stimuli composed of photographs of actors representing characteristics typical of six basic emotions are presented; the participants’ hit rate is assessed

The order in which the instruments were applied was random to avoid bias in data collection. In online collection phase, the participants were recruited via social media (e.g., Facebook, Instagram, Whatsapp) and email. MET was adapted to be applied online on a desktop or mobile using the REDCap platform. Given the REDCap’s limitations to randomize the items, we created two versions of the instrument, i.e., the order in which the items were presented varied. All the instruments described above were applied, except FERT.

The instrument (MET) was reapplied 25 days after the first application (both phases), using the same procedures adopted in the first collection to assess its test-retest reliability.

### Data analysis

The descriptive statistical analyses, normality tests, comparisons between the groups, and correlation analyses were conducted using the Statistical Package for Social Sciences (SPSS-version 23.0). Furthermore, the instrument’s reliability was assessed using Cronbach’s alpha and McDonald’s omega via the Jamovi software (version 1.6.23). Furthermore, we verified whether the instrument fitted the Rasch model and checked for differential item functioning (DIF) according to gender and between MET versions (computer-based (face-to-face) and online) using the WINSTEPS software (version 5.2.3).

Sociodemographic and clinical data were analyzed with descriptive statistics. The following analysis/criteria were adopted for the psychometric study:

a) The instrument’s reliability was assessed using Cronbach’s alpha and McDonald’s omega (values above 0.60 are satisfactory [31,32]. Test-retest reliability was verified using the Intraclass Correlation Coefficient (ICC) and item-total correlation. The correlations’ magnitude was classified according to criteria suggested by Streiner et al [33]: weak (between 0–0.25), moderate (between 0.26–0.50), strong (between 0.51–0.70), and very strong (above 0.71);

b) The model fitted the dichotomous [34] and polytomous Rasch models [35]. Reliability, Person Separation Index and Item Separation Index were assessed. Reliability is expected to be above 0.70, while Separation Indexes above 1.53 indicate the existence of at least two groups of respondents or two different skill levels [36]. Performance deviations were assessed using the infit and outfit indexes. According to Wright [37], variations between 0.5 and 1.5 *logits* are acceptable for infit and outfit. Additionally, the difficulty of the items was estimated using a logit scale [38], with its mean value established at zero, with easier items presenting negative values and more challenging items presenting positive values.

c) The scores were compared between genders (validity based on known groups) using Student’s t-test. Bootstrapping was performed using the bias-corrected and accelerated method with 1000 resamplings for the results to present greater reliability, correct deviations from the normal distribution, and the differences between the groups’ sizes; a 95% confidence interval was established for the differences between means [39]. The variance homogeneity assumption was assessed using the Levene test.

d) The convergent validity study between MET, its subscales, and the IRI was performed using Spearman’s correlation. Considering that both instruments assess the empathy construct, the hypothesis is that the correlation between them is direct and of strong magnitude (between 0.51–0.70). Likewise, the study of convergent validity with correlated constructs (coping (BRCS), stress (PSS), and recognition of facial emotions) was conducted, for which correlations of moderate magnitude are expected (between 0.26–0.50). The correlations’ magnitudes were classified according to the guidelines proposed by Streiner et al [33].

## Results

### Characterization of the samples

The samples’ main sociodemographic and clinical characteristics are presented in Table 1. Note that their profiles are similar regarding the variables highlighted here, which enable comparisons (i.e., most were women, single, aged 32 on average, with a high educational level; approximately 30% reported health problems, and 16% to 27% reported a psychiatric diagnosis).

**Table 1:** Sociodemographic and clinical characterization of the samples.

		Computer-based (face-to-face) N = 142	Online N = 519
Variable		N (%)	N (%)
**Gender**	Female	94 (66.2)	384 (74.0)
**Age (years)**	Mean (SD)	32.41 (14.8)	32.29 (11.8)
**Education**	More than 12 years	88 (62.0)	447 (86.1)
**Children**	Yes	41 (28.9)	123 (23.7)
**Marital status**	No partner	94 (66.2)	443 (61.3)
**Live**	Together	124 (87.3)	367 (85.4)
**Religion**	Yes	106 (74.6)	283 (54.5)
	Praticante	50 (35.2)	236 (45.5)
**Job**	Active	77 (54.2)	334 (64.4)
**Health problem**	Yes	52 (36.6)	164 (31.6)
**Medication use**	Yes	52 (36.6)	201 (38.7)
**Psychiatric diagnosis**	Yes	24 (16.9)	139 (26.8)

### Reliability indicators and item analyses

Regarding item-total correlations, the cognitive subscale items showed weak correlations in both versions (computer-based: 0.23 to 0.29, p<0.05; online: -0.08 to 0.27, p<0.05). However, the opposite was found for the emotional subscale in both versions (face-to-face: 0.31 to 0.75, p<0.05; online: 0.28 to 0.76, p<0.05).

Regarding the reliability indicators, Cronbach’s alpha and McDonald’s omega values were below the expected for the cognitive subscale (face-to-face: α = 0.50, ω = 0.53; online: α = 0.42; ω = 0.46). On the other hand, the results for the emotional subscale were excellent (face-to-face: α = 0.96, ω = 0.96; online: α = 0.96, ω = 0.97). The cognitive subscale obtained moderate reliability coefficients in the test-retest (face-to-face: ICC (95% CI) = 0.49 (0.20–0.72)—for items: ICC = 0.22–0.57; online: ICC (95%CI) = 0.59 (0.47–0.69)—for items: ICC = 0.26–0.82. As for the emotional subscale, the coefficients’ magnitudes varied between strong and very strong (face-to-face: ICC (95% CI) = 0.97 (0.95–0.98)–for the items ICC = 0.34–0.85; online: ICC (95%CI) = 0.98 (0.97–0.99)–for the items: ICC = 0.61–0.87. Further details are provided in the S2 Table.

The average percentage of correct answers provided to the cognitive subscale was 66.12% for the computer-based (face-to-face) version and 67.73% for the online version, while the mean score obtained in the emotional empathy subscale was 6.19 (SD = 0. 53) for the computer-based (face-to-face) version and 6.46 (SD = 0.83) for the online version. The mean scores of these indices were similar between the two formats. S3 Table presents this information in detail.

Person-Item Maps are presented in (S2–S5 Figs). Tables 2 and 3 present the reliability, *Person/ Item Separation Index*, difficulty estimates, and performance deviations (infit and outfit) for the cognitive and emotional subscales of the computer-based (face-to-face) and online versions.

**Table 2:** Estimates of difficulty, performance deviations, reliability and Person/ Item Separation Index for the MET cognitive subscale.

**Stimulus**	**Item**	**Valence**	**MET Computer-based version**				**MET Online version**			
			**Difficulty**	**SE**	**Infit**	**Outfit**	**Difficulty**	**SE**	**Infit**	**Outfit**
1	Agonized	N	1.01	0.17	1.09	1.09	0.36	0.09	1.04	1.05
2	Agonized	N	0.56	0.17	0.99	0.98	1.15	0.09	0.99	0.98
3	Fearful	N	-1.56	0.29	0.95	0.80	-1.42	0.16	0.97	1.00
4	Appalled	N	0.09	0.18	0.98	0.98	0.54	0.09	0.97	0.97
5	Stunned	N	1.01	0.17	1.00	1.03	1.23	0.09	0.98	0.98
6	Crestfallen	N	1.99	0.20	1.10	1.23	3.34	0.15	1.03	2.06
7	Dejected	N	-1.48	0.28	1.01	1.11	-1.82	0.18	0.97	0.97
8	Grief-stricken	N	1.84	0.19	1.13	1.23	2.34	0.11	1.02	1.07
9	Despaired	N	-0.50	0.20	1.02	1.04	-0.72	0.12	0.97	0.92
10	Hopeless	N	-0.01	0.18	1.02	1.01	-0.21	0.11	0.95	0.89
11	Disillusioned	N	0.92	0.17	1.04	1.06	0.61	0.09	0.96	0.95
12	Agonized	N	-0.01	0.18	0.91	0.87	-0.18	0.10	1.01	1.01
13	Pained	N	0.47	0.18	1.02	1.01	0.53	0.09	1.03	1.04
14	Weary	N	0.62	0.17	0.98	0.98	0.35	0.09	1.03	1.03
15	Frustraded	N	-0.97	0.23	1.08	1.24	-1.10	0.14	1.03	1.09
16	Heartbroken	N	0.65	0.17	0.95	0.93	-0.16	0.10	0.99	0.97
17	Intimidated	N	0.47	0.18	0.93	0.92	0.72	0.09	1.03	1.04
18	Pensive	N	-2.32	0.39	0.96	0.73	-1.45	0.16	0.99	1.01
19	Pleading	N	0.41	0.18	0.93	0.91	0.49	0.09	0.96	0.94
20	Sad	N	0.44	0.18	0.95	0.94	0.49	0.09	0.99	0.98
21	Animated	P	-0.67	0.21	1.01	0.98	-0.50	0.11	1.00	1.00
22	Loving	P	-0.46	0.20	1.03	1.03	-0.03	0.10	1.01	1.01
23	Contemplative	P	0.56	0.17	0.96	0.96	0.07	0.10	0.99	1.01
24	Cheerful	P	-0.67	0.21	0.93	0.84	-0.13	0.10	1.00	0.99
25	Carefree	P	1.13	0.18	0.92	0.90	0.36	0.09	1.04	1.06
26	Amused	P	2.80	0.26	1.02	1.20	3.17	0.14	1.03	1.03
27	Adoring	P	0.86	0.17	1.09	1.12	1.05	0.09	1.07	1.08
28	Euphoric	P	-1.40	0.27	0.95	0.82	-1.60	0.17	0.94	0.79
29	Excited	P	1.26	0.18	1.06	1.08	2.10	0.10	1.01	1.04
30	Joyful	P	-0.38	0.20	0.98	0.97	-0.51	0.11	1.00	0.96
31	Grateful	P	-0.30	0.19	1.00	1.00	-1.45	0.16	1.01	1.06
32	Interested	P	0.53	0.17	0.97	0.97	1.13	0.09	1.01	1.01
33	Nostalgic	P	0.02	0.18	1.04	1.05	0.29	0.10	0.99	0.98
34	Satisfied	P	-0.05	0.19	0.96	0.95	-0.47	0.11	0.98	0.94
35	Satisfied	P	-1.33	0.26	0.99	0.94	-1.06	0.14	0.97	0.88
36	Relaxed	P	-1.56	0.29	0.98	0.92	-1.63	0.17	0.98	0.91
37	Content	P	-1.14	0.25	1.00	0.94	-1.78	0.18	0.99	0.97
38	Shy	P	1.13	0.18	1.08	1.10	1.01	0.09	1.04	1.04
39	Triumphant	P	-1.93	0.33	0.98	1.11	-2.26	0.22	0.94	0.62
40	Victorious	P	-2.05	0.35	0.93	0.67	-2.82	0.28	0.97	0.99
			**MET Computer-based version**				**MET Online version**			
Person reliability			0.47				0.40			
Item reliability			0.96				0.99			
Person Separation Index			0.94				0.81			
Item Separation Index			5.12				10.43			

SE = Standard error; P = Positive; N = Negative.

**Table 3:** Difficulty estimates, performance deviations, reliability and Person/ Item Separation Index for the MET emotional subscale.

**Stimulus**	**Item**	**Valence**	**MET Computer-based version**				**MET Online version**			
			**Difficulty**	**SE**	**Infit**	**Outfit**	**Difficulty**	**SE**	**Infit**	**Outfit**
1	Agonized	N	-0.06	0.05	1.23	1.07	-0.28	0.03	1.26	1.09
2	Agonized	N	-0.06	0.05	1.20	1.01	-0.20	0.03	1.27	1.03
3	Fearful	N	0.05	0.05	1.08	0.96	-0.18	0.03	1.47	1.22
4	Appalled	N	0.28	0.04	1.04	2.22	0.45	0.02	0.87	1.18
5	Stunned	N	0.29	0.04	0.84	0.83	0.22	0.02	0.98	1.07
6	Crestfallen	N	0.16	0.04	0.69	0.67	0.14	0.02	0.67	0.63
7	Dejected	N	0.01	0.05	0.85	0.78	0.02	0.03	0.90	0.88
8	Grief-stricken	N	0.17	0.04	0.63	0.78	0.14	0.02	0.67	0.66
9	Despaired	N	-0.02	0.05	1.12	0.99	-0.19	0.03	1.33	1.08
10	Hopeless	N	0.06	0.05	0.74	0.72	0.07	0.02	0.79	0.74
11	Disillusioned	N	0.10	0.05	0.81	0.76	-0.12	0.03	1.11	1.03
12	Agonized	N	0.17	0.04	0.85	1.65	0.12	0.02	1.12	1.17
13	Pained	N	0.02	0.05	0.89	0.86	-0.01	0.03	0.86	0.85
14	Weary	N	0.14	0.04	0.96	0.88	-0.03	0.03	0.97	0.93
15	Frustraded	N	0.04	0.05	0.88	0.89	0.09	0.02	0.96	0.91
16	Heartbroken	N	-0.11	0.05	1.33	1.14	-0.40	0.03	1.51	1.14
17	Intimidated	N	0.01	0.05	0.95	0.93	-0.27	0.03	1.33	1.09
18	Pensive	N	0.07	0.05	0.72	0.64	0.08	0.02	0.90	0.96
19	Pleading	N	-0.11	0.05	1.35	1.18	-0.14	0.03	1.33	1.09
20	Sad	N	-0.04	0.05	0.90	0.78	-0.24	0.03	1.09	0.91
21	Animated	P	-0.07	0.05	1.02	1.00	0.07	0.02	0.77	0.73
22	Loving	P	0.02	0.05	1.09	1.07	0.03	0.02	0.99	0.93
23	Contemplative	P	0.16	0.04	0.80	0.83	0.13	0.02	0.71	0.67
24	Cheerful	P	-0.28	0.05	1.04	0.95	-0.24	0.03	0.96	0.89
25	Carefree	P	-0.30	0.05	1.47	1.29	-0.07	0.03	1.02	1.18
26	Amused	P	0.06	0.05	0.95	0.87	0.17	0.02	1.14	1.24
27	Adoring	P	0.17	0.04	1.07	1.12	0.17	0.02	0.90	0.86
28	Euphoric	P	0.00	0.05	1.18	1.31	0.25	0.02	1.37	1.79
29	Excited	P	-0.04	0.05	1.29	2.09	-0.05	0.03	0.98	0.91
30	Joyful	P	-0.41	0.06	1.09	0.99	-0.35	0.03	1.19	1.04
31	Grateful	P	-0.55	0.06	1.36	1.33	-0.49	0.03	1.09	0.89
32	Interested	P	0.24	0.04	1.06	1.09	0.19	0.02	0.93	0.85
33	Nostalgic	P	0.14	0.04	0.97	0.99	0.20	0.02	0.88	0.90
34	Satisfied	P	-0.18	0.05	1.15	1.03	-0.05	0.03	1.02	0.98
35	Satisfied	P	0.06	0.05	1.13	1.14	0.26	0.02	1.06	1.36
36	Relaxed	P	0.14	0.04	1.03	1.00	0.14	0.02	1.28	1.51
37	Content	P	0.05	0.05	0.88	0.88	0.15	0.02	1.06	1.04
38	Shy	P	-0.08	0.05	1.08	0.99	0.08	0.02	0.92	0.83
39	Triumphant	P	-0.19	0.05	1.01	1.16	-0.04	0.03	1.23	1.24
40	Victorious	P	-0.09	0.05	0.99	1.13	0.19	0.02	1.31	1.67
			**MET Computer-based version**				**MET Online version**			
Person reliability			0.91				0.92			
Item reliability			0.92				0.98			
Person Separation Index			3.15				3.45			
Item Separation Index			3.37				7.28			

Table 2 shows that persons’ reliability was low in both cognitive subscales, indicating low data reproducibility. Consequently, the Person Separation Index was also below the expected, indicating the samples were homogeneous regarding the skills estimated. The reliability of the cognitive items in both versions showed a good data range, indicating that the items covered different difficulty levels.

Regarding the response pattern to the cognitive items, the computer-based (face-to-face) version did not identify items with a response pattern different than expected. As for the online version, only item 6 (Crestfallen) presented performance deviation (outfit = 2.06).

Table 3 shows high-reliability rates for the emotional subscale in both versions, indicating good data reproducibility. However, the analysis of performance deviations regarding the answers to the emotional items revealed three items with outfits outside the parameters (items 4, 12, and 29) in the computer-based (face-to-face) version and two in the online version (items 28 and 40). These findings suggest unexpected response patterns among people with latent trait levels different from these items’ difficulty levels.

### Validity indicators

As expected, the known-group validity analysis revealed that women obtained statistically higher means in the computer-based (face-to-face) version’s cognitive and emotional subscales. However, the results obtained in the online version indicate that women obtained higher means only in the emotional subscale. Further details are available in S4 Table.

Table 4 shows the MET’s convergent validity indicators of both the computer-based (face-to-face) and online versions. Only low/ moderate magnitude correlations were found between the IRI and its subscales (except Personal Distress) with the emotional subscales of the MET in the online version. In the computer-based (face-to-face) version, these correlations were found only for the Perspective Taking subscale.

**Table 4:** MET convergent/ correlated construct validity indicators.

			MET Computer-based version						MET Online version				
External variables	Cog	Cog +	Cog -	A	A +	A -	Cog	Cog +		Cog -	A	A+	A -
**IRI–Total**	0.05	0.09	-0.01	0.04	0.07	0.03	0.04	0.00		0.04	0.31*	0.21*	0.36*
**IRI–EC**	0.11	0.13	0.04	0.09	0.05	0.11	0.06	-0.01		0.07	0.34*	0.26*	0.38*
**IRI–PT**	0.05	0.07	0.01	0.17*	0.15*	0.17*	0.01	0.01		0.00	0.28*	0.23*	0.29*
**IRI–PD**	-0.07	0.00	-0.10	-0.10	0.01	-0.15	0.02	0.01		0.02	0.01	-0.06	0.06
***Resilient Coping***	-0.04	-0.09	0.02	0.19*	0.21*	0.16	0.01	0.04		-0.02	0.12*	0.23*	0.02
**Perceived Stress**	-0.03	0.02	-0.05	-0.27*	-0.29*	-0.19*	0.01	-0.03		0.04	-0.05	-0.22*	0.09
**FERT–Hit Rate–total**	0.21*	0.31*	0.03	0.18*	0.12	0.16	-	-		-	-	-	-
**FERT–Hit Rate—Happiness**	0.16	0.20*	0.07	0.02	0.02	0.07	-	-		-	-	-	-
**FERT–Hit Rate—Sadness**	0.15	0.16	0.02	0.14	0.01	0.16	-	-		-	-	-	-
**FERT–Hit Rate—Fear**	0.05	0.04	0.01	0.24*	0.18*	0.23*	-	-		-	-	-	-
**FERT–Hit Rate—Disgusted**	0.03	0.16	-0.12	-0.04	-0.12	-0.08	-	-		-	-	-	-
**FERT–Hit Rate—Anger**	0.22*	0.28*	0.10	0.04	0.18*	-0.03	-	-		-	-	-	-
**FERT–Hit Rate—Surprise**	0.03	0.00	0.07	0.18*	0.04	0.19*	-	-		-	-	-	-

A = Emotional Subscale; A + = Positive Emotional Subscale; A— = Negative Emotional Subscale; Cog = Cognitive Subscale; Cog + = Positive Cognitive Subscale; Cog— = Negative Cognitive Subscale; EC = Empathic Concern; FERT = Facial Emotion Recognition Task; PD = Personal Distress; PT = Perspective Taking; * Statistically significant correlation (p<0.05).

A low magnitude correlation was found between resilient coping and the total / positive emotional subscale in the both version of the MET. As for perceived stress, low and moderate magnitude correlations were found, specially with the emotional subscale in computer-based (face-to-face) version. Regarding TREF, low and moderate correlations were found between the percentage of correct answers concerning happiness, fear, anger, surprise, and some of the MET’s specific subscales.

Regarding the correlations involving the MET subscales, a strong association was observed between the positive and negative subscales and the total (cognitive: r >0.73; emotional: r >0.77), in both versions. The correlation between the positive and negative subscales was less expressive (r = 0.21–0.25) and not significant when considering the emotional and cognitive subscales

## Discussion

This study presents psychometric indicators concerning the reliability and validity of the MET’s computer-based (face-to-face) and online versions adapted to Brazil. Therefore, to minimize bias, two convenient samples with similar sociodemographic and clinical profiles were addressed.

Regarding reliability, satisfactory internal consistency was found for the emotional subscale, corroborating the moderate to strong correlations between most items and the instrument’s total score. Nonetheless, most of the item-total correlations of the cognitive subscale were weak, harming its internal consistency; the set of items presented low covariance with the total score, with a limited predictive ability [40].

Different psychometric studies involving MET have recurrently presented reliability indicators lower than expected for the cognitive subscale [17,21]. Müller [21] notes that this may be related to the dichotomous characteristic of this set of items. However, this hypothesis is weakened when the indicators verified in other studies using dichotomous scoring instruments, i.e., the Early Trauma Inventory Self Report—Short Form [41] and the Haj-Yahia’s Questionnaire [42] are considered because these proved appropriate.

In agreement with our findings, the Cronbach’s alphas reported for the American version [17 was 0.49 for the cognitive subscale and 0.94 for the emotional subscale. Therefore, in line with the literature [32,43], these authors proposed excluding the items presenting a low correlation with the total. Hence, a version with 19 (out of the 40 initial items) remained; this shorter version did not impact alpha (0.51). The authors considered that a potential explanation is related to some items’ ceiling effect, possibly restricting the test’s inter-individual accuracy range.

Items presenting low correlations with the total score were not excluded in this study because most items presented weak correlations, and analyses indicated that none of the items would increase alpha values if they were excluded. As for the hypothesis proposed by Foell et al. [17] regarding the ceiling effect (items with a percentage of correct answers above 80%), a large number of items with a ceiling effect were found (MET’s computer-based version: 10 items– 25.0% of the total; MET’s online version: 15 items– 37.5% of the total). However, this hypothesis cannot be fully confirmed since items that did not show a ceiling effect also presented a weak item-total correlation. Additionally, at an exploratory level, internal consistency was analyzed by excluding these items, and the indices remained inadequate (<0.43).

Therefore, these findings suggest that more global aspects related to the empathy construct and how it is measured, from the cognitive perspective, may be associated with the cognitive subscale’s poor psychometric performance. Studies adopting other empathy instruments, such as the Empathy Assessment Index [44] and The Basic Empathy Scale [45], also found that the cognitive subscales’ indicators are below those for the emotional subscale, which seems to support this hypothesis. As discussed below, this subscale also presents other weak psychometric indicators (e.g., convergent validity). In contrast, indicators resulting from specific analyses of items (infit and outfit) are satisfactory and do not justify the removal of any item.

With regard to test-retest reliability, temporal stability of the emotional subscale is observed. For the cognitive subscale, less robust values were observed for some individual items (six items with values below 0.30) and for the subscale as a whole (0.49–0.59). Thus, although these general indices are acceptable, there is a certain weakness in relation to this psychometric quality. In the study by Yu [16] carried out with the Chinese version, in an interval of 28 days, the test-retest reliability was adequate for both subscales: 84 for the cognitive and 0.80 for the emotional.

Rasch analysis of the cognitive and emotional items indicated high reliability in both versions (>0.92). This finding suggests that the item difficulty hierarchy tends to be reproduced in future surveys [36]. On the other hand, person reliability concerning skill levels was adequate only for the emotional items (>0.91). It was below the expected for the cognitive items, suggesting that we cannot affirm that the participants’ skill estimates concerning the cognitive items will be reproduced in future surveys.

This fact is possibly associated with the small skill range found in the sample for these items; it was concentrated between 0 and 2 logits for both versions. According to Linacre [36], the lack of a sufficiently large skill range (ideally between -3 and 3 logits) negatively impacts reliability indicators, considering that not all possible skill levels are represented in the sample and considered in the analysis. It is noteworthy, however, that the reliability obtained via Rasch analysis does not refer to data quality but rather to the reproducibility or non-reproducibility of the parameters found here [36].

Regarding the distribution of the items’ difficulty level, the analyzes indicated that, in general, the skill interval between -1 and 1 was the best represented by the test, indicating that people with a latent trait level within this range are possibly more accurately evaluated than people with a higher and/or lower latent trait level [36]. Such a fact may negatively impact the instrument’s ability to discriminate between individuals, especially those with extremely high or low skill levels.

Differences were also found in the scores obtained by men and women in both subscales, indicating validity evidence. Thus, MET discriminated between these known groups, reinforcing its clinical validity, considering the vast literature indicating that women present higher levels of empathy than men [12,46–48]. According to Brody [49], these differences between genders are due to sociocultural variables, especially regarding how girls and boys are socialized. While girls are encouraged to express affection and care, boys are taught to inhibit pro-social behavior. Eisenberg and Lennon [50] consider that social desirability may have a potential impact, leading the female group to endorse items more frequently/intensively. Differences at the biological level, such as in hormone concentrations (e.g., testosterone [51]) and some brain regions’ functioning patterns [52], also possibly explain such differences.

Potential relationships regarding the convergent validity parameters between the MET and the IRI were investigated. IRI is also an instrument with an internal structure composed of domains linked to cognitive empathy (Perspective Taking) and emotional empathy (Personal Distress and Empathic Concern). Contrary to expectations, the cognitive subscale was not associated with any IRI subscale; Dziobek et al. [15] and Foell et al. [17] had already reported this finding. Yu [16] found significant correlations but a very low magnitude (0.14). Correlations between the MET and IRI emotional subscales were significant but low in magnitude and only for the Empathic Concern and Personal Distress subscales. These findings reinforce the results reported by previous studies [15–17], in which weak associations were predominant (0.63, 0.22, and 0.15, respectively).

These results reinforce an intriguing question in the field related to the low convergent validity of instruments addressing empathy. Lima and Osório [12] consider that the lack of consensus on how the construct can be represented and assessed impacts psychometric indicators. For instance, the cognitive aspects of empathy in the IRI are assessed with sentences such as *“I try to look at everybody’s side of a disagreement before I make a decision*.*”* On the other hand, identifying emotions in the MET’s cognitive subscale [15] is more related to recognizing facial expressions and to the Theory of Mind, which involves recognizing other peoples’ mental states [53].

Lima and Osório [12] reviewed the factor structure of more than a dozen empathy instruments and verified a lack of a common theoretical model to adequately explain the instruments’ internal structure. They consider that it probably contributes to this lack of convergence among the instruments, requiring a deeper discussion about the construct.

Ding et al. [13] note that the fact that the relationships between self-report empathy scales and empathic behavior tasks are weak to moderate should not be considered a problem because these are not redundant but potentially complementary measures. These observations conceivably explain the indicators found in this study, reinforcing the use of multi-method assessments to minimize disadvantages between different methodologies [14].

Regarding the results involving the recognition of facial emotions, this construct is considered one of the domains that compose social cognition, sharing common characteristics with empathic skills, such as the ability to identify details in other people’s behaviors and signify experienced phenomena [53]. Therefore, the low magnitude correlations evidenced by MET suggest convergence between these constructs that represent domains of correlated social cognition. Such a fact had previously been reported by Parreira [54] when studying the IRI and Empathy Quotient [55], which showed correlations between 0.16 and 0.28 in a facial recognition task of basic emotions.

On the other hand, associations between empathy measures addressing clinically relevant correlates, such as aggressiveness, alexithymia, and impulsiveness, among others [12], are well documented. Associations with a resilience measure and a measure of perceived stress were tested in this study. Unlike the study conducted by Vinayak and Judge [56], in which the correlations between empathy (Toronto Empathy Questionnaire [57]) and resilience (Conner-Davidson Resilience Scale [58]) were strong (0.67), in this study the correlations with resilience were significant, but little expressive. The same pattern of correlations was found for the relationship with perceived stress, in line with Gupta and Kiran [59], in which only weak/moderate correlations (r = 0.28) were found between the scores of emotional empathy of Basic Empathy Scale [60] and perceived stress.

In general, the MET showed adequate psychometric indicators, in line with previous studies, which supports its use in research and clinical practice. Its innovative format stands out, with greater ecological validity and possibility of use in different formats. The emotional subscale performed better, while some weaknesses of the cognitive subscale should be mentioned, especially from the perspective of the classical test theory. These weaknesses need to be further explored by addressing larger samples to ensure more robust conclusions regarding whether it is adequate. In addition, future studies addressing the instrument’s internal structure can contribute to this understanding.

Later, studies that test the invariance of responses to the MET as a function of the type of application (computer-based/face-to-face and online) are opportune. This is because, according to Luxton et al [61], even in the presence of empirical evidence that a given measure can be applied using technology, there are no guarantees that it will remain compatible. Factors such as performing the test in a context other than the experimental one and changes in the layout of the instrument’s presentation can influence the response pattern, which needs to be analyzed using specific statistical techniques.

Finally, one of the limitations of this study concerns sampling biases related to sample convenience and their respective sociodemographic characteristics, considering that most participants were young adults with high education and skills (restricted range), which must be considered in the interpretation and generalization of data. Future studies should include more heterogeneous samples in relation to these issues and expand the sources and forms (randomized) of recruitment, in order to minimize the impacts of selection bias. Furthermore, it is important to highlight that despite the time adopted for the retest being in accordance with that recommended by the literature, the effect of a possible memory bias cannot be ruled out.

## Conclusions

In general, the MET presented favorable indices regarding temporal stability, convergent validity with measures of empathy (albeit of low magnitude), clinical validity and adequate discrimination capacity. Its items have different levels of difficulty, and this is the first study to demonstrate these indicators. In addition to the well-known computerized version, the version developed for online use also showed good psychometric indicators and can be used as an alternative version, expanding its applicability. The better performance of the emotional subscale and the need for further studies on the cognitive subscale are highlighted.

## Supporting information

S1 Fig Flowchart regarding the inclusion/exclusion of participants in the computer-based (face-to-face) and online version samples. (DOCX)

S2 Fig Item-person map of the MET cognitive subscale–computer-based (face-to-face) version. (DOCX)

S3 Fig Item-person map of the MET emotional subscale–computer-based (face-to-face) version. (DOCX)

S4 Fig Item-person map of the MET cognitive subscale–online version. (DOCX)

S5 Fig Item-person map of the MET emotional subscale–online version. (DOCX)

S1 Table Additional information on data collection. (DOCX)

S2 Table Item-total correlation, internal consistency and test-retest reliability of the MET–computer-based (face-to-face) and online version. * When applying the retest, this stimulus showed zero variance and therefore the coefficient was not obtained; P = Positive; N = Negative.
(DOCX)

S3 Table Indicators related to the items of the cognitive and emotional subscales of the MET–computer-based (face-to-face) and online version, depending on emotional valence. P = Positive; N = Negative.
(DOCX)

S4 Table Indicators of validity based on external measures—known groups—MET cognitive and emotional subscales–computer-based (face-to-face) and online version. CI = Confidence interval; df = degrees of freedom; SD = Standard deviation.
(DOCX)

S5 Table Analysis of metric invariance between the computer-based (face-to-face) and online versions of the MET considering the release of factor loadings for each item. P = Positive; N = Negative.
(DOCX)
