# Supplementary material for: Multifaceted Empathy Test (MET): Validity evidence for the Brazilian population concerning the computer-based (face-to-face) and online versions
Source: PLoS One. 2023 Jul 13;18(7):e0284524. doi: 10.1371/journal.pone.0284524 (PMC10343083; doi:10.1371/journal.pone.0284524)
Supplement: S1 Fig — (DOCX) [file pone.0284524.s001.docx]

S1 Fig. Flowchart regarding the inclusion/exclusion of participants in the computer-based (face-to-face) and online version samples.

**
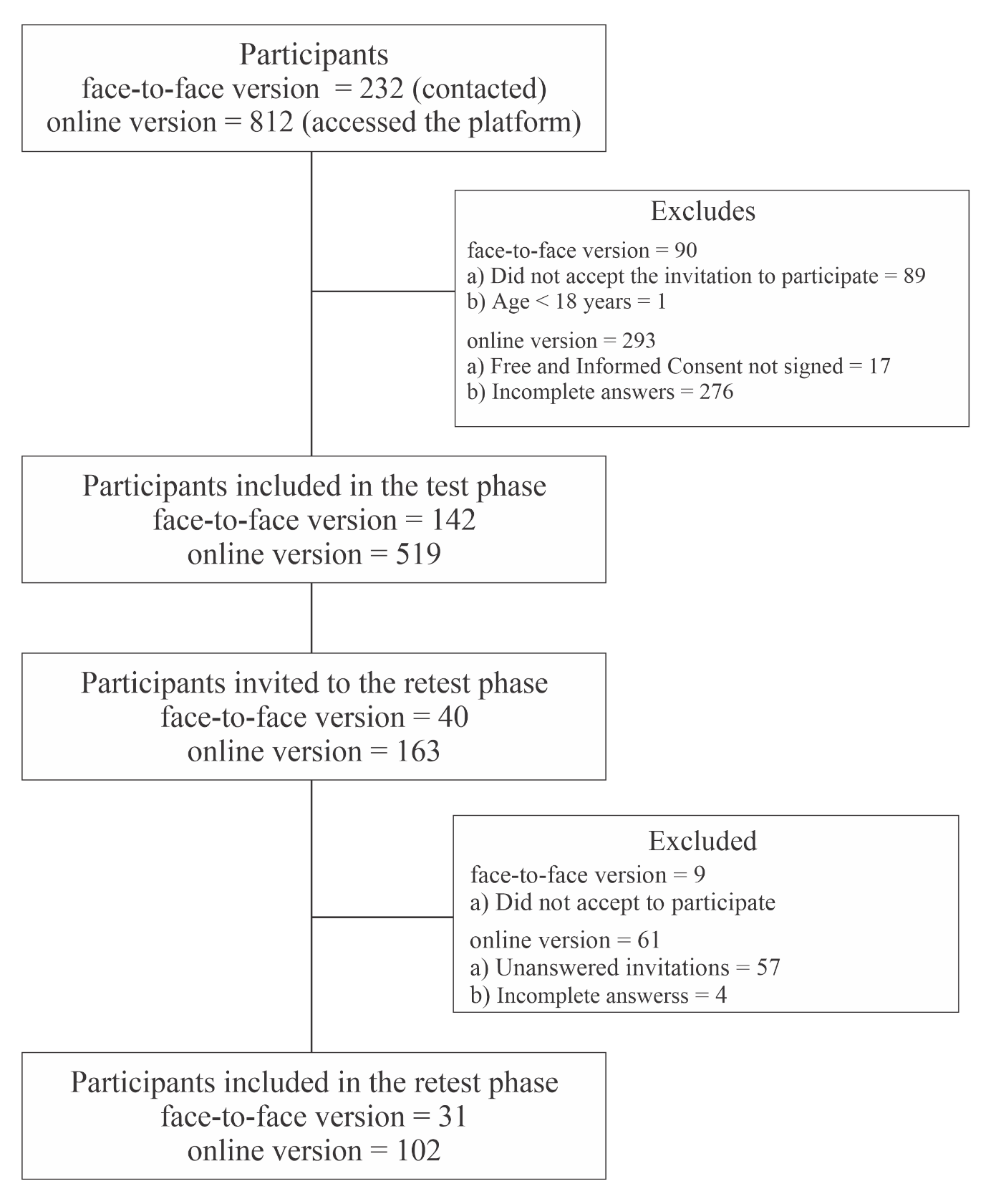
**
