# Supplementary material for: Multifaceted Empathy Test (MET): Validity evidence for the Brazilian population concerning the computer-based (face-to-face) and online versions
Source: PLoS One. 2023 Jul 13;18(7):e0284524. doi: 10.1371/journal.pone.0284524 (PMC10343083; doi:10.1371/journal.pone.0284524)
Supplement: S5 Fig — (DOCX) [file pone.0284524.s005.docx]

S5 Fig. Item-Person Map of the MET Emotional Subscale – online version

P = Positive; N = Negative; E = Emotional
