# Supplementary material for: Multifaceted Empathy Test (MET): Validity evidence for the Brazilian population concerning the computer-based (face-to-face) and online versions
Source: PLoS One. 2023 Jul 13;18(7):e0284524. doi: 10.1371/journal.pone.0284524 (PMC10343083; doi:10.1371/journal.pone.0284524)
Supplement: S1 Table — (DOCX) [file pone.0284524.s006.docx]

S1 Table. Additional Information on Data Collection

**TIMELINE**

| **Period** | **Activity** | **Participantes** |
| --- | --- | --- |
| July 2020 | Start of online data collection | 812 accesses to the REdcap platform |
| 2nd half July 2020 | Start of online data collection retest | 163 invitations sent by email |
| May 2021 | Start of computerized data collection (face-to-face) | 232 participants contacted |
| 2nd half May 2021 | Start of the retest of computerized data collection (face-to-face) | 142 invited participants (all included in the test phase) |
| July 2021 | End of online data collection | 519 participants included |
| 2nd half July 2021 | End of online retest | 102 participants included |
| October 2021 | End of computerized data collection (face-to-face) | 142 participants included |
| 2nd half October 2021 | End of computerized collection retest (face-to-face) | 31 participants included |

Comments:

1 - Participants did not receive any kind of incentive to participate in the study

2 - Computerized data collection was carried out at the Research Laboratory of the University, in an individual room, with temperature, light and sound control.

3 - In the computerized/face-to-face collection, the data from the computerized instruments were saved directly in a computer and then transferred to the spreadsheet of the statistical program, in which the data from the instruments applied in pencil and paper were entered manually and checked.

4 - in online data collection, all data were automatically saved on the Redcap Platform and then transferred to the statistical program spreadsheet.
