# Supplementary material for: Multifaceted Empathy Test (MET): Validity evidence for the Brazilian population concerning the computer-based (face-to-face) and online versions
Source: PLoS One. 2023 Jul 13;18(7):e0284524. doi: 10.1371/journal.pone.0284524 (PMC10343083; doi:10.1371/journal.pone.0284524)
Supplement: S2 Table — * When applying the retest, this stimulus showed zero variance and therefore the coefficient was not obtained; P = Positive; N = Negative. (DOCX) [file pone.0284524.s007.docx]

| S2 Table. Item-total correlation, internal consistency and test-retest reliability of the MET – Computer-based (face-to-face) and online version | | | | | | | | | | |
| --- | --- | --- | --- | --- | --- | --- | --- | --- | --- | --- |
| **Stimulus** | **Item** | **Valence** | **MET Computer-based version** | | | | **MET Online version** | | | |
|  |  |  | **Cognitive Empathy** | | **Emotional Empathy** | | **Cognitive Empathy** | | **Emotional Empathy** | |
|  |  |  | **Item-total correlation** | **Test-retest reliability** | **Item-total correlation** | **Test-retest reliability** | **Item-total correlation** | **Test-retest reliability** | **Item-total correlation** | **Test-retest reliability** |
| 1 | Agonized | N | -0,04 | 0,49 | 0,66 | 0,54 | 0,01 | 0,46 | 0,61 | 0,71 |
| 2 | Agonized | N | 0,14 | 0,34 | 0,72 | 0,66 | 0,12 | 0,40 | 0,64 | 0,73 |
| 3 | Fearful | N | 0,22 | 0,25 | 0,75 | 0,73 | 0,13 | 0,45 | 0,61 | 0,77 |
| 4 | Appalled | N | 0,16 | 0,42 | 0,66 | 0,72 | 0,15 | 0,67 | 0,28 | 0,74 |
| 5 | Stunned | N | 0,10 | 0,31 | 0,71 | 0,75 | 0,12 | 0,58 | 0,61 | 0,70 |
| 6 | Crestfallen | N | -0,14 | 0,49 | 0,70 | 0,71 | -0,08 | 0,42 | 0,76 | 0,72 |
| 7 | Dejected | N | 0,04 | 0,33 | 0,69 | 0,70 | 0,13 | 0,50 | 0,65 | 0,76 |
| 8 | Grief-stricken | N | -0,16 | 0,55 | 0,72 | 0,79 | 0,01 | 0,65 | 0,72 | 0,76 |
| 9 | Despaired | N | 0,07 | 0,34 | 0,71 | 0,34 | 0,17 | 0,45 | 0,64 | 0,76 |
| 10 | Hopeless | N | 0,09 | 0,52 | 0,70 | 0,79 | 0,22 | 0,60 | 0,70 | 0,69 |
| 11 | Disillusioned | N | 0,04 | 0,57 | 0,72 | 0,83 | 0,18 | 0,59 | 0,63 | 0,76 |
| 12 | Agonized | N | 0,31 | 0,54 | 0,73 | 0,73 | 0,08 | 0,50 | 0,62 | 0,81 |
| 13 | Pained | N | 0,09 | 0,34 | 0,67 | 0,85 | 0,05 | 0,65 | 0,71 | 0,61 |
| 14 | Weary | N | 0,16 | 0,44 | 0,67 | 0,71 | 0,03 | 0,76 | 0,68 | 0,71 |
| 15 | Frustraded | N | -0,10 | 0,43 | 0,65 | 0,72 | 0,00 | 0,56 | 0,66 | 0,68 |
| 16 | Heartbroken | N | 0,23 | 0,42 | 0,67 | 0,69 | 0,12 | 0,51 | 0,61 | 0,75 |
| 17 | Intimidated | N | 0,27 | 0,34 | 0,67 | 0,75 | 0,03 | 0,70 | 0,58 | 0,78 |
| 18 | Pensive | N | 0,20 | * | 0,69 | 0,74 | 0,10 | 0,26 | 0,66 | 0,72 |
| 19 | Pleading | N | 0,27 | 0,40 | 0,66 | 0,70 | 0,18 | 0,63 | 0,63 | 0,71 |
| 20 | Sad | N | 0,21 | 0,32 | 0,73 | 0,75 | 0,12 | 0,53 | 0,63 | 0,71 |
| 21 | Animated | P | 0,09 | 0,33 | 0,50 | 0,69 | 0,09 | 0,62 | 0,68 | 0,83 |
| 22 | Loving | P | 0,05 | 0,57 | 0,43 | 0,71 | 0,08 | 0,43 | 0,63 | 0,84 |
| 23 | Contemplative | P | 0,20 | 0,55 | 0,64 | 0,74 | 0,11 | 0,61 | 0,71 | 0,75 |
| 24 | Cheerful | P | 0,28 | 0,24 | 0,41 | 0,73 | 0,09 | 0,46 | 0,65 | 0,83 |
| 25 | Carefree | P | 0,27 | 0,40 | 0,37 | 0,72 | 0,01 | 0,39 | 0,63 | 0,79 |
| 26 | Amused | P | -0,01 | 0,49 | 0,52 | 0,74 | 0,02 | 0,56 | 0,58 | 0,76 |
| 27 | Adoring | P | -0,07 | 0,41 | 0,48 | 0,73 | -0,05 | 0,59 | 0,66 | 0,82 |
| 28 | Euphoric | P | 0,23 | * | 0,42 | 0,64 | 0,24 | 0,35 | 0,55 | 0,85 |
| 29 | Excited | P | -0,01 | 0,52 | 0,41 | 0,66 | 0,04 | 0,60 | 0,65 | 0,79 |
| 30 | Joyful | P | 0,15 | 0,45 | 0,39 | 0,65 | 0,11 | 0,51 | 0,62 | 0,74 |
| 31 | Grateful | P | 0,12 | 0,49 | 0,31 | 0,73 | 0,03 | 0,56 | 0,63 | 0,73 |
| 32 | Interested | P | 0,19 | 0,45 | 0,48 | 0,78 | 0,07 | 0,49 | 0,65 | 0,86 |
| 33 | Nostalgic | P | 0,04 | 0,47 | 0,56 | 0,79 | 0,12 | 0,64 | 0,69 | 0,84 |
| 34 | Satisfied | P | 0,20 | 0,49 | 0,42 | 0,71 | 0,15 | 0,29 | 0,63 | 0,87 |
| 35 | Satisfied | P | 0,12 | -0,03 | 0,36 | 0,61 | 0,18 | 0,61 | 0,59 | 0,85 |
| 36 | Relaxed | P | 0,15 | 0,52 | 0,54 | 0,70 | 0,12 | 0,74 | 0,56 | 0,75 |
| 37 | Content | P | 0,11 | 0,22 | 0,54 | 0,77 | 0,09 | 0,66 | 0,61 | 0,80 |
| 38 | Shy | P | -0,05 | 0,38 | 0,46 | 0,77 | 0,02 | 0,82 | 0,65 | 0,83 |
| 39 | Triumphant | P | 0,11 | 0,49 | 0,49 | 0,72 | 0,27 | 0,56 | 0,60 | 0,81 |
| 40 | Victorious | P | 0,28 | 0,41 | 0,47 | 0,85 | 0,09 | * | 0,57 | 0,85 |
| * When applying the retest, this stimulus showed zero variance and therefore the coefficient was not obtained; P = Positive; N = Negative | | | | | | | | | | |
