# Supplementary material for: Multifaceted Empathy Test (MET): Validity evidence for the Brazilian population concerning the computer-based (face-to-face) and online versions
Source: PLoS One. 2023 Jul 13;18(7):e0284524. doi: 10.1371/journal.pone.0284524 (PMC10343083; doi:10.1371/journal.pone.0284524)
Supplement: S3 Table — P = Positive; N = Negative. (DOCX) [file pone.0284524.s008.docx]

| S3 Table. Indicators related to the items of the cognitive and emotional subscales of the MET – computer-based (face-to-face) and online version, depending on emotional valence | | | | | | |
| --- | --- | --- | --- | --- | --- | --- |
| **Stimulus** | **Item** | **Valence** | **MET Computer-based version** | | **MET Online version** | |
|  |  |  | **% of hits Cognitive Empathy** | **Mean (SD) Emotional Empathy** | **% of hits Cognitive Empathy** | **Mean (SD) Emotional**  **Empathy** |
| 1 | Agonized | N | 44,40 | 6,42 (2,64) | 64,50 | 7,32 (2,16) |
| 2 | Agonized | N | 54,90 | 6,41 (2,74) | 46,40 | 7,11 (2,37) |
| 3 | Fearful | N | 90,10 | 6,06 (2,80) | 90,90 | 7,08 (2,48) |
| 4 | Appalled | N | 65,50 | 5,24 (2,62) | 60,70 | 2,57 (3,12) |
| 5 | Stunned | N | 44,40 | 5,22 (2,53) | 44,50 | 5,85 (2,38) |
| 6 | Crestfallen | N | 23,90 | 5,69 (2,26) | 9,60 | 6,12 (2,40) |
| 7 | Dejected | N | 89,40 | 6,19 (2,36) | 93,60 | 6,53 (2,23) |
| 8 | Grief-stricken | N | 26,80 | 5,64 (2,21) | 22,00 | 6,14 (2,24) |
| 9 | Despaired | N | 76,80 | 6,30 (2,68) | 83,60 | 7,11 (2,40) |
| 10 | Hopeless | N | 67,60 | 6,02 (2,27) | 75,90 | 6,36 (2,29) |
| 11 | Disillusioned | N | 46,50 | 5,90 (2,46) | 59,20 | 6,91 (2,30) |
| 12 | Agonized | N | 67,60 | 5,64 (2,58) | 75,30 | 6,19 (2,53) |
| 13 | Pained | N | 57,00 | 6,15 (2,38) | 60,90 | 6,59 (2,35) |
| 14 | Weary | N | 53,50 | 5,76 (2,52) | 64,90 | 6,68 (2,35) |
| 15 | Frustraded | N | 83,80 | 6,08 (2,31) | 88,10 | 6,30 (2,43) |
| 16 | Heartbroken | N | 52,80 | 6,57 (2,69) | 75,00 | 7,55 (2,18) |
| 17 | Intimidated | N | 57,00 | 6,21 (2,44) | 56,60 | 7,29 (2,15) |
| 18 | Pensive | N | 95,10 | 6,00 (2,22) | 91,10 | 6,32 (2,31) |
| 19 | Pleading | N | 58,50 | 6,59 (2,68) | 61,80 | 6,97 (2,46) |
| 20 | Sad | N | 57,70 | 6,35 (2,47) | 61,80 | 7,21 (2,10) |
| 21 | Animated | P | 79,60 | 6,46 (2,07) | 80,50 | 6,36 (2,19) |
| 22 | Loving | P | 76,10 | 6,16 (2,06) | 72,60 | 6,48 (2,31) |
| 23 | Contemplative | P | 54,90 | 5,70 (2,27) | 70,70 | 6,16 (2,25) |
| 24 | Cheerful | P | 79,60 | 7,04 (1,78) | 74,40 | 7,22 (2,00) |
| 25 | Carefree | P | 41,50 | 7,09 (2,05) | 64,50 | 6,79 (2,24) |
| 26 | Amused | P | 12,70 | 6,03 (2,11) | 11,20 | 6,04 (2,48) |
| 27 | Adoring | P | 47,90 | 5,65 (2,21) | 48,70 | 6,03 (2,40) |
| 28 | Euphoric | P | 88,70 | 6,23 (2,14) | 92,30 | 5,75 (2,68) |
| 29 | Excited | P | 38,70 | 6,35 (2,21) | 26,00 | 6,73 (2,26) |
| 30 | Joyful | P | 74,60 | 7,36 (1,66) | 80,70 | 7,46 (2,03) |
| 31 | Grateful | P | 73,20 | 7,64 (1,64) | 91,10 | 7,71 (1,80) |
| 32 | Interested | P | 55,60 | 5,40 (2,20) | 47,00 | 5,97 (2,43) |
| 33 | Nostalgic | P | 66,90 | 5,76 (2,28) | 66,10 | 5,91 (2,49) |
| 34 | Satisfied | P | 68,30 | 6,78 (1,97) | 80,20 | 6,73 (2,27) |
| 35 | Satisfied | P | 88,80 | 6,03 (1,99) | 87,70 | 5,71 (2,46) |
| 36 | Relaxed | P | 90,10 | 5,76 (2,31) | 92,50 | 6,15 (2,53) |
| 37 | Content | P | 85,90 | 6,07 (2,06) | 93,40 | 6,11 (2,46) |
| 38 | Shy | P | 41,50 | 6,47 (2,04) | 49,70 | 6,34 (2,31) |
| 39 | Triumphant | P | 93,00 | 6,79 (1,96) | 95,80 | 6,68 (2,41) |
| 40 | Victorious | P | 93,70 | 6,52 (1,99) | 97,50 | 5,96 (2,60) |
| **Valence** | Negative |  | 63,28 (17,71) | 6,02 (0,39) | 64,32 (22,02) | 6,51 (1,04) |
|  | Positive |  | 67,56 (22,25) | 6,36 (0,61) | 71,13 (23,95) | 6,41 (0,56) |
| P = Positive; N = Negative | | | | |  |  |
