# Supplementary material for: Multifaceted Empathy Test (MET): Validity evidence for the Brazilian population concerning the computer-based (face-to-face) and online versions
Source: PLoS One. 2023 Jul 13;18(7):e0284524. doi: 10.1371/journal.pone.0284524 (PMC10343083; doi:10.1371/journal.pone.0284524)
Supplement: S4 Table — CI = Confidence interval; df = degrees of freedom; SD = Standard deviation. (DOCX) [file pone.0284524.s009.docx]

| S4 Table. Indicators of validity based on external measures - known groups - MET cognitive and emotional subscales – computer-based (face-to-face) and online version | | | | | | | | |
| --- | --- | --- | --- | --- | --- | --- | --- | --- |
| **MET Computer-based version** | | | | | | | | |
|  | | ***Scores*** | | ***t* test statistics *(Bootstrapping sample)*** | | | | |
| **Subscale** | **Group** | ***Mean*** | ***SD*** | ***t*** | ***df*** | ***p*-value** | **Mean Difference CI (95%)** | |
|  |  |  |  |  |  |  | **Lower limit** | **Upper limit** |
| Emotional | Women | 254,76 | 57,62 | 2,049 | 140 | 0,042 | 0,70 | 39,61 |
|  | Men | 234,60 | 50,92 |  |  |  |  |  |
| Cognitive | Women | 25,68 | 3,71 | 2,858 | 140 | 0,003 | 0,54 | 3,10 |
|  | Men | 23,81 | 3,62 |  |  |  |  |  |
| **MET Online version** | | | | | | | | |
|  | | ***Scores*** | | ***t* test statistics *(Bootstrapping sample)*** | | | | |
| **Subscale** | **Group** | ***Mean*** | ***SD*** | ***t*** | ***df*** | ***p*-value** | **Mean Difference CI (95%)** | |
|  |  |  |  |  |  |  | **Lower limit** | **Upper limit** |
| Emotional | Women | 264,94 | 59,72 | 4,122 | 517 | 0,001 | 13,13 | 36,56 |
|  | Men | 240,23 | 60,45 |  |  |  |  |  |
| Cognitive | Women | 27,26 | 3,24 | 1,926 | 517 | 0,083 | -0,06 | 1,38 |
|  | Men | 26,61 | 3,69 |  |  |  |  |  |

CI = Confidence interval; df = degrees of freedom; SD = Standard deviation
