# Supplementary material for: Multifaceted Empathy Test (MET): Validity evidence for the Brazilian population concerning the computer-based (face-to-face) and online versions
Source: PLoS One. 2023 Jul 13;18(7):e0284524. doi: 10.1371/journal.pone.0284524 (PMC10343083; doi:10.1371/journal.pone.0284524)
Supplement: S5 Table — P = Positive; N = Negative. (DOCX) [file pone.0284524.s010.docx]

| S5 Table. Analysis of metric invariance between the computer-based (face-to-face) and online versions of the MET considering the release of factor loadings for each item | | | | | | | | | | | | |
| --- | --- | --- | --- | --- | --- | --- | --- | --- | --- | --- | --- | --- |
| **Stimulus** | **Item** | **Valence** | **Fit Index** | | | | | | | | | |
|  |  |  | **Emotional subscale** | | | | | **Cognitive subscale** | | | | |
|  |  |  | **x²** | **CFI** | **TLI** | **RMSEA** | **SRMR** | **x²** | **CFI** | **TLI** | **RMSEA** | **SRMR** |
| 1 | Agonized | N | 10712,63 | 0,88 | 0,88 | 0,06 | 0,14 | 10712,60 | 0,88 | 0,88 | 0,06 | 0,14 |
| 2 | Agonized | N | 10711,01 | 0,88 | 0,88 | 0,06 | 0,14 | 10729,50 | 0,88 | 0,88 | 0,06 | 0,14 |
| 3 | Fearful | N | 10705,72 | 0,88 | 0,88 | 0,06 | 0,14 | 10712,50 | 0,88 | 0,88 | 0,06 | 0,14 |
| 4 | Appalled | N | 10717,71 | 0,88 | 0,88 | 0,06 | 0,14 | 10698,70 | 0,88 | 0,88 | 0,06 | 0,14 |
| 5 | Stunned | N | 10714,65 | 0,88 | 0,88 | 0,06 | 0,14 | 10738,10 | 0,88 | 0,88 | 0,06 | 0,14 |
| 6 | Crestfallen | N | 10711,48 | 0,88 | 0,88 | 0,06 | 0,14 | 10726,70 | 0,88 | 0,88 | 0,06 | 0,14 |
| 7 | Dejected | N | 10678,63 | 0,89 | 0,88 | 0,06 | 0,14 | 10741,40 | 0,88 | 0,88 | 0,06 | 0,14 |
| 8 | Grief-stricken | N | 10716,03 | 0,88 | 0,88 | 0,06 | 0,14 | 10720,00 | 0,88 | 0,88 | 0,06 | 0,14 |
| 9 | Despaired | N | 10702,25 | 0,88 | 0,88 | 0,06 | 0,14 | 10744,90 | 0,88 | 0,87 | 0,06 | 0,14 |
| 10 | Hopeless | N | 10711,97 | 0,88 | 0,88 | 0,06 | 0,14 | 10724,10 | 0,88 | 0,88 | 0,06 | 0,14 |
| 11 | Disillusioned | N | 10709,91 | 0,88 | 0,88 | 0,06 | 0,14 | 10720,10 | 0,88 | 0,88 | 0,06 | 0,14 |
| 12 | Agonized | N | 10710,94 | 0,88 | 0,88 | 0,06 | 0,14 | 10725,50 | 0,88 | 0,88 | 0,06 | 0,14 |
| 13 | Pained | N | 10713,31 | 0,88 | 0,88 | 0,06 | 0,14 | 10722,60 | 0,88 | 0,88 | 0,06 | 0,14 |
| 14 | Weary | N | 10712,43 | 0,88 | 0,88 | 0,06 | 0,14 | 10703,30 | 0,88 | 0,88 | 0,06 | 0,14 |
| 15 | Frustraded | N | 10712,02 | 0,88 | 0,88 | 0,06 | 0,14 | 10730,40 | 0,88 | 0,88 | 0,06 | 0,14 |
| 16 | Heartbroken | N | 10709,08 | 0,88 | 0,88 | 0,06 | 0,14 | 10716,30 | 0,88 | 0,88 | 0,06 | 0,14 |
| 17 | Intimidated | N | 10714,00 | 0,88 | 0,88 | 0,06 | 0,14 | 10676,50 | 0,88 | 0,88 | 0,06 | 0,14 |
| 18 | Pensive | N | 10715,26 | 0,88 | 0,88 | 0,06 | 0,14 | 10705,20 | 0,88 | 0,88 | 0,06 | 0,14 |
| 19 | Pleading | N | 10705,88 | 0,88 | 0,88 | 0,06 | 0,14 | 10724,20 | 0,88 | 0,88 | 0,06 | 0,14 |
| 20 | Sad | N | 10679,36 | 0,89 | 0,88 | 0,06 | 0,14 | 10727,60 | 0,88 | 0,88 | 0,06 | 0,14 |
| 21 | Animated | P | 10700,40 | 0,88 | 0,88 | 0,06 | 0,14 | 10729,10 | 0,88 | 0,88 | 0,06 | 0,14 |
| 22 | Loving | P | 10693,68 | 0,89 | 0,88 | 0,06 | 0,14 | 10729,30 | 0,88 | 0,88 | 0,06 | 0,14 |
| 23 | Contemplative | P | 10715,35 | 0,88 | 0,88 | 0,06 | 0,14 | 10730,70 | 0,88 | 0,88 | 0,06 | 0,14 |
| 24 | Cheerful | P | 10712,65 | 0,88 | 0,88 | 0,06 | 0,14 | 10729,40 | 0,88 | 0,88 | 0,06 | 0,14 |
| 25 | Carefree | P | 10715,30 | 0,88 | 0,88 | 0,06 | 0,14 | 10728,80 | 0,88 | 0,88 | 0,06 | 0,14 |
| 26 | Amused | P | 10716,05 | 0,88 | 0,88 | 0,06 | 0,14 | 10736,50 | 0,88 | 0,88 | 0,06 | 0,14 |
| 27 | Adoring | P | 10689,53 | 0,89 | 0,88 | 0,06 | 0,14 | 10711,90 | 0,88 | 0,88 | 0,06 | 0,14 |
| 28 | Euphoric | P | 10721,41 | 0,88 | 0,88 | 0,06 | 0,14 | 10719,30 | 0,88 | 0,88 | 0,06 | 0,14 |
| 29 | Excited | P | 10703,87 | 0,88 | 0,88 | 0,06 | 0,14 | 10726,80 | 0,88 | 0,88 | 0,06 | 0,14 |
| 30 | Joyful | P | 10716,05 | 0,88 | 0,88 | 0,06 | 0,14 | 10729,30 | 0,88 | 0,88 | 0,06 | 0,14 |
| 31 | Grateful | P | 10701,22 | 0,88 | 0,88 | 0,06 | 0,14 | 10740,30 | 0,88 | 0,88 | 0,06 | 0,14 |
| 32 | Interested | P | 10698,23 | 0,89 | 0,88 | 0,06 | 0,14 | 10723,80 | 0,88 | 0,88 | 0,06 | 0,14 |
| 33 | Nostalgic | P | 10705,82 | 0,88 | 0,88 | 0,06 | 0,14 | 10678,60 | 0,88 | 0,88 | 0,06 | 0,14 |
| 34 | Satisfied | P | 10719,97 | 0,88 | 0,88 | 0,06 | 0,14 | 10728,80 | 0,88 | 0,88 | 0,06 | 0,14 |
| 35 | Satisfied | P | 10715,75 | 0,88 | 0,88 | 0,06 | 0,14 | 10741,70 | 0,88 | 0,88 | 0,06 | 0,14 |
| 36 | Relaxed | P | 10720,17 | 0,88 | 0,88 | 0,06 | 0,14 | 10727,50 | 0,88 | 0,88 | 0,06 | 0,14 |
| 37 | Content | P | 10719,58 | 0,88 | 0,88 | 0,06 | 0,14 | 10727,20 | 0,88 | 0,88 | 0,06 | 0,14 |
| 38 | Shy | P | 10692,40 | 0,89 | 0,88 | 0,06 | 0,14 | 10722,90 | 0,88 | 0,88 | 0,06 | 0,14 |
| 39 | Triumphant | P | 10720,79 | 0,88 | 0,88 | 0,06 | 0,14 | 10733,40 | 0,88 | 0,88 | 0,06 | 0,14 |
| 40 | Victorious | P | 10707,67 | 0,88 | 0,88 | 0,06 | 0,14 | 10747,90 | 0,88 | 0,87 | 0,06 | 0,14 |
| P = Positive; N = Negative | | | | | | | | | | | | |
